# The Protective Effects of Chrysin on Acrylamide‐Induced Hepatotoxicity: Insights Into Oxidative Stress, Inflammation, Apoptosis, Autophagy, and Histological Evaluation in Rats

**DOI:** 10.1002/jbt.70334

**Published:** 2025-06-09

**Authors:** Selman Gencer, Nurhan Akaras, Hasan Şimşek, Cihan Gür, Mustafa İleritürk, Sefa Küçükler, Fatih Mehmet Kandemir

**Affiliations:** ^1^ Department of Internal Diseases, Faculty of Medicine Aksaray University Aksaray Turkey; ^2^ Department of Histology and Embryology, Faculty of Medicine Aksaray University Aksaray Turkey; ^3^ Department of Physiology, Faculty of Medicine Aksaray University Aksaray Turkey; ^4^ Department of Medical Laboratory Techniques, Vocational School of Health Services Atatürk University Erzurum Turkey; ^5^ Department of Animal Science, Horasan Vocational College Atatürk University Erzurum Turkey; ^6^ Department of Veterinary Biochemistry, Faculty of Veterinary Atatürk University Erzurum Turkey; ^7^ Department of Medical Biochemistry, Faculty of Medicine Aksaray University Aksaray Turkey

**Keywords:** acrylamide, apoptosis, autophagy, chrysin, hepatotoxicity, inflammation

## Abstract

Acrylamide (ACR) is a toxic chemical with a high carcinogenic risk that is released as a result of heating or processing foods at high temperatures. Chrysin (CHR) is a flavonoid that is naturally found in foods such as honey and passionflower and stands out with its antioxidant, anticancer, and anti‐inflammatory properties. This study aims to determine the protective effects of CHR in ACR‐induced hepatotoxicity. ACR was administered orally at a dose of 38.27 mg/kg; CHR (25 or 50 mg/kg) was administered orally for ten days. Biochemical and molecular methods were used to investigate oxidative stress, inflammation, and apoptotic markers in liver tissue. Additionally, histological methods were used to determine the liver tissue's structural and functional characteristics and autophagy. CHR treatment alleviated ACR‐induced oxidative stress by increasing antioxidants (SOD, CAT, GPx, GSH) and reducing increased oxidant MDA. CHR reduced inflammatory activity by inactivating NF‐κB and pro‐inflammatory cytokines. ACR‐induced increases in apoptotic Casp‐3, Casp‐6, Casp‐9, and Bax were reduced by CHR, while the decreased level of antiapoptotic Bcl‐2 was increased. It was also determined immunohistochemically that CHR inhibited autophagic Beclin‐1 activity. CHR was effective in reducing ACR‐induced hepatotoxicity damage and may be an effective treatment option.

## Summary

- Acrylamide induces hepatotoxicity.
- Chrysin attenuates acrylamide‐induced oxidative stress.
- Chrysin attenuates acrylamide‐induced inflammation.
- Chrysin attenuates acrylamide‐induced apoptosis.
- Chrysin attenuates acrylamide‐induced autophagy.

## Graphical abstract

CHR exhibited significant protective effects against ACR‐induced hepatotoxicity by reducing oxidative stress, decreasing inflammation and apoptosis, and inhibiting autophagic activity, highlighting its potential as an effective therapeutic agent.

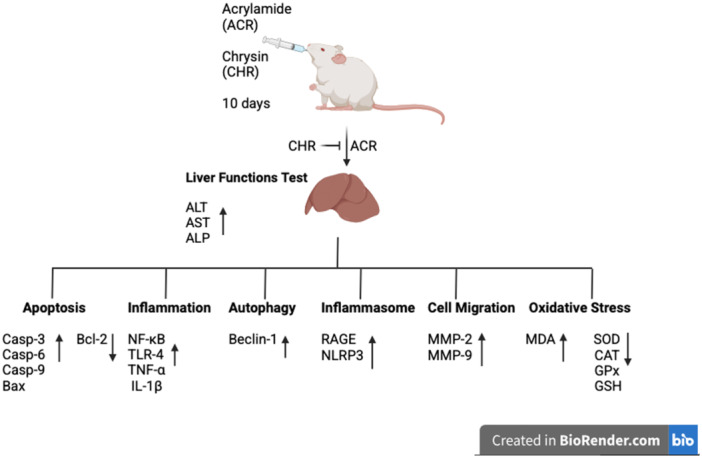

## 1 Introduction

Acrylamide (ACR) is a toxic chemical released during the heating and processing of food, causing global concern [1]. ACR is used in the treatment of drinking water, cosmetics, and mining, as well as being released during the heat treatment of fried products such as potatoes and bread as a significant source of exposure [2]. Another significant source of ACR is cigarette smoke [3]. Heat‐induced reactions between the amino group of the free amino acid asparagine and the carbonyl groups of glucose and fructose in foods mostly release ACR [4].

ACR was identified as a potential carcinogen by the International Agency for Research on Cancer in 1994, for reasons such as its toxic effect after it is metabolized and penetrates all organs and tissues in the body [5]. ACR exposure has been evaluated in different populations, and in April 2002, the Swedish National Food Authority and Stockholm University jointly announced that the light processing of carbohydrate‐rich foods releases significant levels of ACR. In particular, an essential amount of ACR of 2300 μg/kg (2.3 PPM) was reported in French fries and chips. In general, the body can withstand an average exposure of 4 µg/kg bw/day, but ACR can cause tissue toxicity in humans at 200 µg/kg bw/day and carcinogenicity at 300 µg/kg bw/day [6].

Since ACR is easily soluble in water, it can be rapidly absorbed by the body and penetrate tissues. Once absorbed by the body, it can bind to DNA through a Michael addition‐type reaction and exert toxic and carcinogenic effects [7]. The oxidative biotransformation of ACR is carried out by the enzyme cytochrome P450 (CYP2E1), which converts it to glycidamide. This conversion of ACR causes an increase in oxidative stress and may cause toxic effects in vital organs such as the liver, kidney, and brain [8].

Liver tissue is the main organ that metabolizes toxins, environmental pollutants, chemicals, and drugs that need to be removed from the body. Therefore, dysfunction in the metabolizing pathways in the liver can cause damage to liver tissue, leading to many liver diseases [9]. Oxidative stress is one of the primary mechanisms in ACR hepatoxicity. ACR‐induced reactive oxygen species (ROS) production can adversely affect cell survival due to oxidative degradation of lipids, proteins, and cell membrane damage due to irreversible DNA modification [10]. Studies have reported that ACR causes tissue toxicity through oxidative stress and also alters enzyme activities [11].

The use of plant‐derived phytochemicals has come to the forefront against toxic agents affecting liver and kidney tissues. Flavonoids, which are naturally found in almost all plants, are polyphenolic phytochemicals [12]. Flavonoids contribute to human health as they have important biological activities. The average daily intake of flavonoids with a normal diet is estimated to be around 1–2 g per day [13]. Chrysin (CHR) is a natural flavonoid found in honey, propolis, fruits, vegetables, and passion fruit (*Passiflora sp*.) [14, 15]. CHR was found to be 0.10–5.3 mg/kg in different kinds of honey and as high as 28 g/L in propolis [16]. CHR has important biological and pharmacological activities such as antioxidant activity, anti‐inflammatory, anticancer, and antihypertension [17]. CHR has been reported to provide significant protection against toxic damage in different organs in In‐Vivo studies [18]. CHR increases the activity of significant antioxidant enzymes such as glutathione peroxidase (GPx) and glutathione (GSH) in cancer models and reduces the effect of cytochrome P450 (CytP450)‐dependent monooxygenases [19]. Due to enterohepatic reabsorption, CHR can be recycled and reused in the intestine, resulting in increased local bioavailability [20]. Chemically, CHR has two benzene rings (i.e., rings A and B) and a six‐membered heterocyclic ring (i.e., ring C). Unlike flavonoids, CHR contains only hydroxyl groups on ring A (5,7‐dihydroxyl) and no substituents on ring B. This unique substituent feature renders it a suitable model compound for studying structure–activity relationships. The pharmacological activity of CHR is related to the moieties on rings A and C, where the hydroxyl may exert antitoxic effects. However, CHR is safe to use in preclinical models. Daily consumption of 0.5–3.0 g of CHR is safe for humans [21].

The present study aimed to determine the level of ACR‐induced toxic damage in rat liver tissue and the ameliorative effect of CHR through different damage pathways.

## 2 Materials and Methods

### 2.1 Chemicals

ACR (Sigma, CAS Number 79‐06‐1, molecular weight: 71.08 g/mol) and CHR (Sigma, cas no: 480‐40‐0, purity 97%) were used in the experimental procedures. All other chemicals used in the analysis were obtained from Sigma and Merck.

### 2.2 Experimental Groups

In the present study, thirty‐five male Wistar rats (12 weeks old, 230–250 g) were obtained from KONUDAM (Konya/Turkey). The rats were housed under standard laboratory conditions (24°C–25°C, 45%–50% humidity, and a 12/12 h light/dark cycle). Rats were fed with standard rat chow (Supporting Information) and drinking water ad libitum. The rats were divided into groups for 3 days for adaptation and started to be housed in the area where the treatments would be performed. The rats were randomly divided into five groups with seven animals in each group.

1. Control (C): For 10 days, saline was administered orally once daily.
2. Acrylamide (ACR): ACR at a dose of 38.27 mg/kg was administered orally once daily for 10 days.
3. Chrysin (CHR): CHR is administered orally at 50 mg/kg once daily for 10 days.
4. Acrylamide + Chrysin‐25 (ACR + CHR25): ACR at a dose of 38.27 mg/kg orally once daily for 10 days. After 30 min, CHR 25 mg/kg orally once daily for 10 days.
5. Acrylamide + Chrysin‐50 (ACR + CHR50): ACR at a dose of 38.27 mg/kg orally once daily for 10 days. After 30 min, CHR 50 mg/kg orally once a day for 10 days.

Literature was used to determine doses, application methods, and the number of animals used in groups [22, 23]. ACR was dissolved in saline (1 mL) while CHR was dissolved in corn oil (0.5 ml). Doses were prepared daily. The reported LD50 for ACR and CHR are 114.71 mg/kg [24] and 4350 mg/kg [25], respectively, and our administered doses represented approximately 33.7% of these LD50s for ACR and 0.5%–1.0% for CHR.

All experimental procedures were carried out in accordance with the European Directive 2010/63/EU for animal experiments. All procedures performed in this study involving animals complied with the ARRIVE guidelines.

### 2.3 Tissue Collection

Twenty‐four hours after the last treatment (Day 11), the animals were decapitated under light sevoflurane anesthesia, and liver and blood samples were collected. Blood samples were taken from the jugular vein. Blood samples were transferred into vacuum tubes without anticoagulant for biochemical analyses, centrifuged at 3000 rpm at +4°C for 10 min, separated into sera, and stored in a deep freezer at −20°C until biochemical analyses were performed. Some of the liver tissues were taken for biochemical analyses and stored at −20°C until the analyses were performed. The other part was placed in a 10% formaldehyde solution for histologic examinations.

### 2.4 Liver Function Tests

To determine liver function, serum alanine aminotransferase (ALT), aspartate aminotransferase (AST), and alkaline phosphatase (ALP) activities were determined using an enzyme‐linked immunosorbent assay (ELISA) reader (Bio‐Tek, Winooski, VT, USA) and commercial kits (TML Diagnostic Medical Products, Ankara, Turkey) as previously performed in our laboratory [26].

### 2.5 Oxidative Stress Analysis

To determine the oxidative stress level of liver tissues, the level of malondialdehyde (MDA), an indicator of lipid peroxidation, as an oxidant, and the activities of superoxide dismutase (SOD), catalase (CAT) and GPx, and GSH level as antioxidants were analyzed. For this purpose, liver tissues were treated with liquid nitrogen and ground in a mortar and pestle. The ground tissues were then mixed with 1.15% potassium chloride (KCl) (1:10 ratio) and homogenized using a homogenizer (Tissue Lyser II, Qiagen, The Netherlands). After the homogenates were centrifuged, the supernatants were used. For SOD activity Sun et al. [27] method; CAT activity by the Aebi [28] method; GPx activity by Lawrence and Burk [29] method; GSH level by Sedlak and Lindsay [30] method; and MDA levels by Placer et al. [31]. The total protein content of liver tissues was analyzed by the Lowry et al. [32] method.

### 2.6 Real Time‐PCR (RT‐PCR)

Total RNAs were isolated from rat liver tissues using QIAzol Lysis Reagent (79306; Qiagen). Then, cDNAs were synthesized from RNAs with iScript cDNA Synthesis Kit (1708891, Bio‐Rad). The obtained cDNAs were analyzed for Nuclear factor kappa B (NF‐κB), Toll‐like receptor 4 (TLR‐4), Tumor necrosis factor‐alpha (TNF‐α), Interleukin‐1 beta (IL‐1β), Receptors for advanced glycation end products (RAGE), NLR family pyrin domain containing 3 (NLRP3), Cysteine‐aspartic proteases‐3 (Casp‐3), Cysteine‐aspartic proteases‐6 (Casp‐6), Cysteine‐aspartic proteases‐9 (Casp‐9), BCL2 Associated X (Bax), B‐cell lymphoma 2 (Bcl‐2), matrix metalloproteinase‐2 (MMP‐2), and matrix metalloproteinase‐9 (MMP‐9) primers and iTaq Universal SYBR Green Supermix (172‐5121, BIO‐RAD) were mixed and reacted in Rotor‐Gene Q (Qiagen). After the reaction cycles were completed, genes were normalized to β‐Actin by the ${2^{‐\Delta \Delta C}}_{T}$method [33]. The sequences of all genes are presented in Table 1.

**Table 1:** Primer sequences.

Gene	Sequences (5′‐3′)	Length (bp)	Accession no
NF‐κB	F: AGTCCCGCCCCTTCTAAAAC R: CAATGGCCTCTGTGTAGCCC	106	NM_001276711.1
TLR‐4	F: GCTCTGCCAAGTCTCAGATA R: GCTCTTCTAGACCCATGAAG	160	NM_019178.2
TNF‐α	F: CTCGAGTGACAAGCCCGTAG R: ATCTGCTGGTACCACCAGTT	139	NM_012675.3
IL‐1β	F: ATGGCAACTGTCCCTGAACT R: AGTGACACTGCCTTCCTGAA	197	NM_031512.2
RAGE	F: CTGAGGTAGGGCATGAGGATG R: TTCATCACCGGTTTCTGTGACC	113	NM_053336.2
NLRP3	F: TCCTGCAGAGCCTACAGTTG R: GGCTTGCAGCACTGAAGAAC	185	NM_001191642.1
Caspase‐3	F: ACTGGAATGTCAGCTCGCAA R: GCAGTAGTCGCCTCTGAAGA	270	NM_012922.2
Caspase‐6	F: AGACCTTGACTGGCTTGTTCA R: TCTGTCTGATGATCCACCACG	139	NM_001271984.1
Caspase‐9	F: ACGTGAACTTCTGCCCTTCC R: GGTCGTTCTTCACCTCCACC	117	NM_031632.3
Bax	F: TTTCATCCAGGATCGAGCAG R: AATCATCCTCTGCAGCTCCA	154	NM_017059.2
Bcl‐2	F: GACTTTGCAGAGATGTCCAG R: TCAGGTACTCAGTCATCCAC	214	NM_016993.2
MMP2	F: CTCTAGGAGAAGGACAAGTG R: CTCAAAGTTGTACGTGGTGG	158	NM_031054.2
MMP9	F: AGCTGGCAGAGGATTACCTG R: ATGATGGTGCCACTTGAGGT	230	NM_031055.2
β‐Actin	F: CAGCCTTCCTTCCTGGGTATG R: AGCTCAGTAACAGTCCGCCT	360	NM_031144.3

### 2.7 Hematoxylin and Eosin Staining

Liver tissue samples were kept in 10% formalin solution for 72 h for fixation. Tissue samples were passed through alcohol and xylene series using standard tissue processing procedures and embedded in paraffin blocks. Then, preparations were obtained by taking five micron thick sections using a microtome. Preparations were stained with hematoxylin‐eosin (H&E), and the stained tissues were evaluated and photographed using a light microscope (Olympus Cx43; Japan). A semiquantitative histopathological scoring was performed evaluating three criteria: pyknosis, vascular congestion, and mononuclear cell infiltration in liver tissue. The results were evaluated as negative (0), mild (1), moderate (2) and severe (3) using a blinded method according to histopathological changes under light microscopy [34, 35].

### 2.8 Immunohistochemical Analysis

Three micrometers thick sections taken from liver tissues were prepared for staining after being passed through xylene and alcohol series. Antigen retrieval was performed by keeping the sections in ethylene diamine tetra acetic acid (EDTA) buffer. Then, it was kept in 3% hydrogen peroxide for 10 min. The sections, in which endogenous peroxidase activity was inhibited, were washed with phosphate‐buffered saline (PBS) and then treated with protein block for 10 min. The primary antibody (beclin‐1; Invitrogen, PA1‐16857) diluted with PBS was dropped onto the sections and kept in the refrigerator (+4°C) overnight. Then, after washing with PBS three times for 5 min, they were treated with secondary antibody and Strepto Biotin, respectively. After each procedure, 3,3ʹ‐diaminobenzidine (DAB) solution was dropped on the sections washed with PBS and waited until a brown color appeared. The sections were treated with Harris Hematoxylin for 5 min and washed again with PBS. The tissues were kept in 96% alcohol for 5 min, in absolute alcohol twice for 5 min, and in xylene twice for 5 min and then covered with entellan. The sections were evaluated as negative (0), mild (1), moderate (2) and severe (3) according to their immune positivity. The criteria of < 10% positive cells (1), 10%–50% positive cells (2) and > 50% positive cells (3) were used in scoring [36, 37]. Five sections were randomly selected from each group and the staining intensity was evaluated with ImageJ software (Image J, version 1.46a, NIH, Bethesda, MD, USA).

### 2.9 Statistical Analysis

SPSS 20.0 software (IBM, Chicago, IL) was used for data analysis. Statistical analysis results were expressed as mean ± standard deviation. Tukey's post hoc test and one‐way analysis of variance (ANOVA) were used for multiple comparisons of the data. Significance levels were determined at three different levels (*p* < 0.05, *p* < 0.01, and *p* < 0.001).

## 3 Results

### 3.1 Liver Function Test Results

ALT, AST, and ALP activities were determined from liver tissues to define the level of liver function (Table 2). ACR administration increased ALT, AST, and ALP activities (*p* < 0.001). When CHR was administered together with ACR, it decreased ALT, AST, and ALP activities at both doses (*p* < 0.001), affecting the effect of ACR in the opposite direction. CHR was more effective at 50 mg/kg dose when dose comparison was made (*p* < 0.001).

**Table 2:** Effect of CHR on hepatic serum markers and oxidative stress biomarkers in ACR‐induced hepatotoxicity.

Parameters	Control	CHR	ACR	ACR + CHR 25	ACR + CHR 50
ALP (U/L)	73.43 ± 8.14	65.71 ± 6.99^###^	166.57 ± 12.35***	125.86 ± 10.68***^/###/✩✩✩^	88.57 ± 7.89*^/###^
ALT(U/L)	28.86 ± 4.02	28.14 ± 3.53^###^	109.57 ± 1.36***	75.14 ± 6.47***^/###/✩✩✩^	45.14 ± 3.80**^/###^
AST (U/L)	65.86 ± 8.63	65.14 ± 6.77^###^	225.00 ± 16.17***	170.86 ± 12.69***^/###/✩✩✩^	90.57 ± 9.34**^/###^
MDA(nmol/g tissue)	38.74 ± 3.81	34.32 ± 3.05^###^	103.45 ± 8.20***	78.04 ± 4.87***^/###/✩✩✩^	53.04 ± 5.03***^/###^
GSH (nmol/g tissue)	7.11 ± 0.31	7.31 ± 0.33^###^	2.18 ± 0.10***	3.34 ± 0.18***^/###/✩✩✩^	5.02 ± 0.25***^/###^
CAT (catal/g protein)	40.77 ± 3.60	40.18 ± 3.14^###^	13.80 ± 1.83***	19.23 ± 1.93***^/##/✩✩✩^	32.90 ± 2.56***^/###^
SOD (U/g protein)	28.54 ± 2.81	28.50 ± 2.43^###^	8.13 ± 1.30***	14.64 ± 1.79***^/###/✩✩✩^	20.91 ± 2.02***^/###^
GPx (U/g protein)	29.61 ± 2.67	30.44 ± 2.40^###^	9.99 ± 1.46***	13.10 ± 1.91***^/✩✩✩^	18.84 ± 2.41***^/###^

*Note:* Statistical significance (control vs. others: *p < 0.05, **p < 0.01, ***p < 0.001, ACR vs. others: ##*p* < 0.01, ###*p* < 0.001, ACR + CHR 25 vs. ACR + CHR 50: ^✩✩✩^ *p* < 0.001) was analyzed using One Way ANOVA.

### 3.2 Oxidative Stress Results

MDA level as oxidant, GSH level as antioxidant, and SOD, CAT, and GPx activities were determined in liver tissues (Table 2). ACR administration increased MDA level and decreased antioxidants (GSH, SOD, CAT, and GPx) (*p* < 0.001). When CHR was applied together with ACR, the oxidant MDA level reduced, while the antioxidant levels increased. This change was *p* < 0.001 in MDA, GSH and SOD, *p* < 0.01 in CAT, and not significant in GPx in the CHR25 group. In the CHR50 group, the change in all parameters was at *p* < 0.001 significance level. When CHR dose comparison was made, 50 mg/kg dose was more effective on all parameters (*p* < 0.001).

### 3.3 Inflammation Results

**Figure 1:**
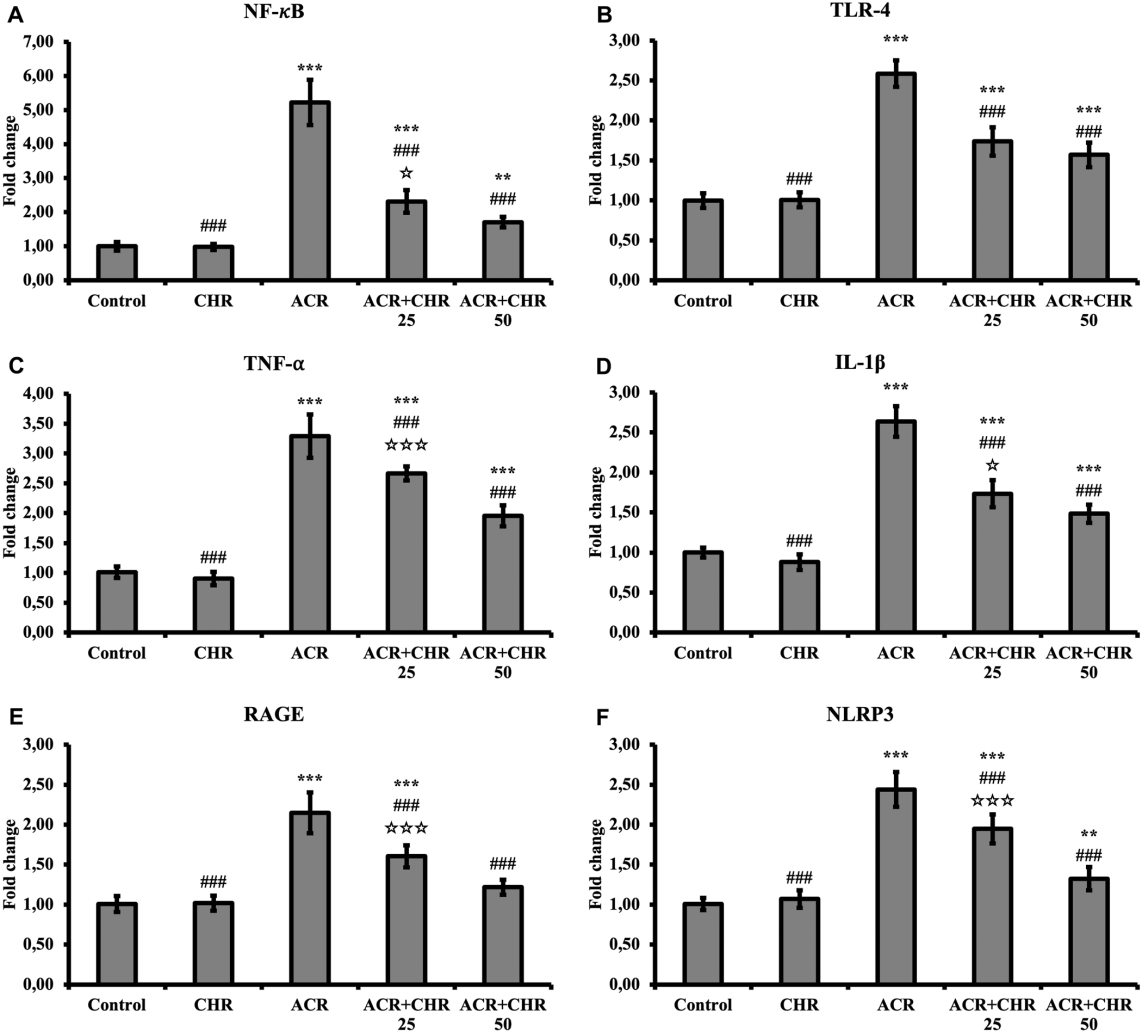
Effects of ACR and CHR administrations on NF‐κB (A), TLR‐4 (B), TNF‐α (C), IL‐1β (D), RAGE (E) and NLRP3 (F) mRNA transcription levels in liver tissue of rats. Values are given as mean ± SD. Control versus others: *p < 0.05, **p < 0.01, ***p < 0.001, ACR versus others: #*p *< 0.05, ##*p *< 0.01, ###*p *< 0.001, ACR + CHR 25 versus ACR + CHR 50: ✩*p* < 0.05, ✩✩*p *< 0.01, ✩✩✩*p *< 0.001.

NF‐κB, TLR‐4, TNF‐α, and IL‐1β mRNA transcription levels were determined from liver tissues to determine the level of inflammation damage (Figure 1). ACR increased all of these inflammatory parameters (*p* < 0.001). When CHR was co‐administered with ACR, it decreased inflammatory parameters at both doses (*p* < 0.001), affecting the effect of ACR in the opposite direction. In the dose comparison, 50 mg/kg CHR was determined to be more effective (NF‐κB and IL‐1β: *p* < 0.05; TNF‐α: *p* < 0.001, TLR‐4: no difference).

### 3.4 Inflammasome Results

RAGE and NLRP3 mRNA transcription levels were determined in liver tissues for the detection of inflammasome levels (Figure 1). ACR increased RAGE and NLRP3 levels (*p* < 0.001). CHR treatment attenuated the effect of ACR and decreased RAGE and NLRP3 levels (both doses: *p* < 0.001). In the dose comparison, 50 mg/kg CHR was determined to be more effective.

### 3.5 Apoptosis Results

**Figure 2:**
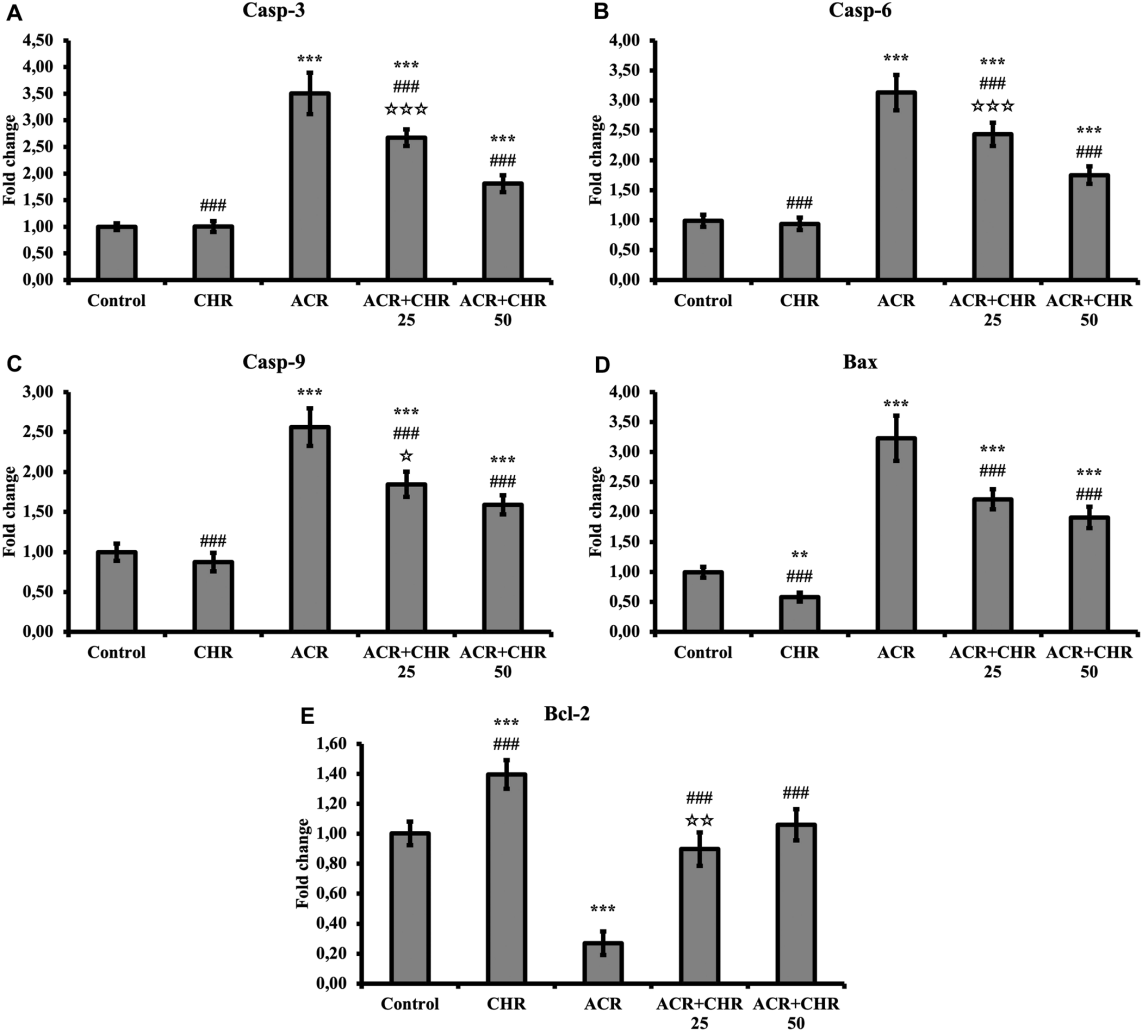
Effects of ACR and CHR administrations on Casp‐3 (A), Casp‐6 (B), Casp‐9 (C), Bax (D), and Bcl‐2 (E) mRNA transcription levels in liver tissue of rats. Values are given as mean ± SD. Control versus others: *p < 0.05, **p < 0.01, ***p < 0.001, ACR versus others: #*p *< 0.05, ##*p *< 0.01, ###*p *< 0.001, ACR + CHR 25 versus ACR + CHR 50: ✩*p* < 0.05, ✩✩*p *< 0.01, ✩✩✩*p *< 0.001.

Apoptotic Casp‐3, Casp‐6, Casp‐9, and Bax and antiapoptotic Bcl‐2 mRNA transcription levels were determined to determine the level of apoptotic damage in liver tissues (Figure 2). ACR increased the level of apoptotics and decreased the level of antiapoptotic Bcl‐2 (*p* < 0.001). Since CHR was applied together with ACR, CHR decreased the level of apoptotics and increased the level of antiapoptotic Bcl‐2 (*p* < 0.001). In the dose comparison, 50 mg/kg CHR was determined to be more effective (Casp‐3 and Casp‐6: *p* < 0.001; Casp‐9: *p* < 0.05; Bcl‐2; *p* < 0.01, Bax: no difference).

### 3.6 Cell Migration Results

**Figure 3:**
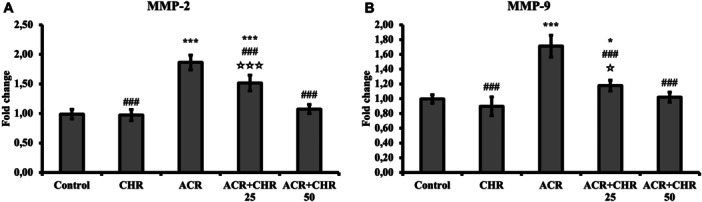
Effects of ACR and CHR administrations on MMP‐2 and MMP‐9 mRNA transcription levels in liver tissue of rats. Values are given as mean ± SD. Control versus others: *p < 0.05, **p < 0.01, ***p < 0.001, ACR versus others: #*p *< 0.05, ##*p *< 0.01, ###*p *< 0.001, ACR + CHR 25 versus ACR + CHR 50: ✩*p* < 0.05, ✩✩*p *< 0.01, ✩✩✩*p *< 0.001.

MMP‐2 and MMP‐9 mRNA transcription levels were determined for cell migration in liver tissues (Figure 3). MMP‐2 and MMP‐9 mRNA transcription levels increased in the ACR‐treated group (*p* < 0.001). This effect of ACR was reversed with CHR administration and CHR decreased MMP‐2 and MMP‐9 levels (*p* < 0.001). In the dose comparison, 50 mg/kg CHR was determined to be more effective (MMP‐2: *p* < 0.001; MMP‐9: *p* < 0.05).

### 3.7 H&E Results

**Figure 4:**
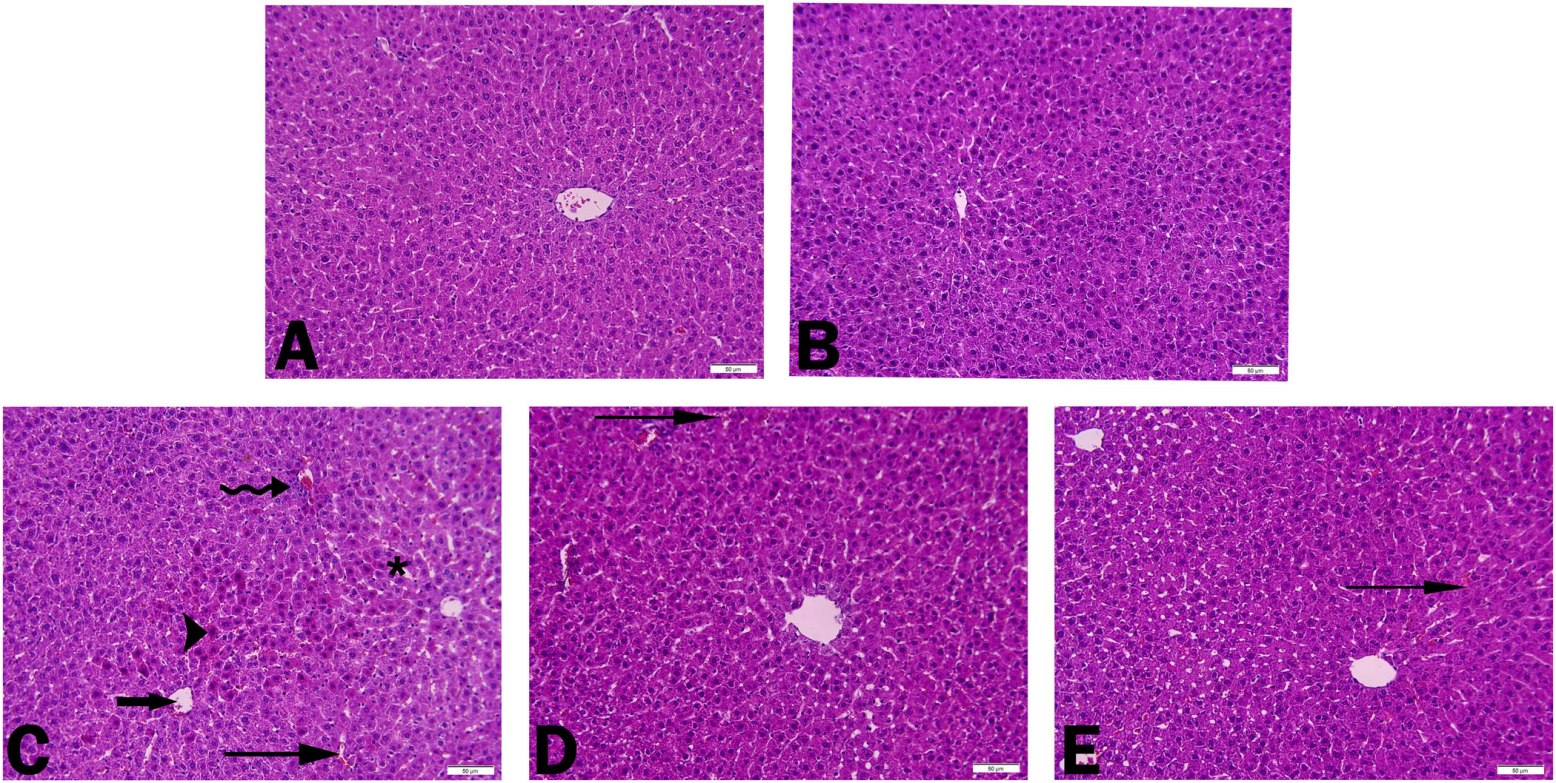
Histopathological examination of rat liver tissues treated with ACR and CHR. Regular histological appearance of control (A) and CHR (B) treated liver tissues. Appearance of hepatocyte cells with eosinophilic stained pyknotic nuclei (arrowhead), central vein congestion (thick arrow), sinusoidal congestion (thin arrow), mononuclear cell infiltration (curved arrow), sinusoidal dilatation (star) in the livers of rats treated with ACR (C). ACR + CHR 25 (D) and ACR + CHR 50 (E) groups show mild congestion in sinusoidal vessels (thin arrow), H&E, Bar: 50 μm.

Histological examinations revealed no histological difference between the control and CHR groups. Therefore, the control group was taken as basis and evaluations were made accordingly. When the liver tissues of the control and CHR‐only groups were examined, it was observed that hepatocytes with smooth cell borders extending from the central vein and classical hepatic lobules were observed (Figure 4A,B). In the ACR group, hepatocytes with pyknotic nuclei and hydropic degeneration were noted. Additionally, ACR administration caused mononuclear cell infiltration, sinusoidal dilatation and congestion in vessels and sinusoids (Figure 4C). In the experimental groups given CHR together with ACR, striking histological changes were detected in parallel with the dose increase. Mild congestion was detected in the interstitial vessels in the ACR + CHR25 and ACR + CHR 50 groups (Figure 4D,E). As a result, the liver was significantly protected in the CHR‐treated groups compared to the ACR‐treated group. Histopathological findings are summarized in Table 3.

**Table 3:** Histopathological and immunohistochemical findings and scores in rat liver tissue.

Parameters	Control	CHR	ACR	ACR + CHR 25	ACR + CHR 50
Pyknosis	0.14 ± 0.37^#^	0.14 ± 0.37	2.14 ± 0.37*	0.42 ± 0.53^#^	0.28 ± 0.48^#^
Vascular congestion	0.14 ± 0.37^#^	0.14 ± 0.37	2.42 ± 0.53*	1.00 ± 0.57*^#^	0.85 ± 0.37*^#^
Mononuclear cell infiltration	0.00^#^	0.00	1.85 ± 0.37*	0.57 ± 0.53*^#^	0.14 ± 0.37^#+^
Beclin‐1 expression	0.00^#^	0.00	2.14 ± 0.37*	1.00 ± 0.57*^#^	0.85 ± 0.37*^#^

*Note:* Data are mean ± SD. *(*p* < 0.05) compared to control groups, ^#^(*p* < 0.05) compared to ACR group, ^+^(*p* < 0.05) compared to ACR + CHR 25 group.

### 3.8 Immunohistochemical Results

**Figure 5:**
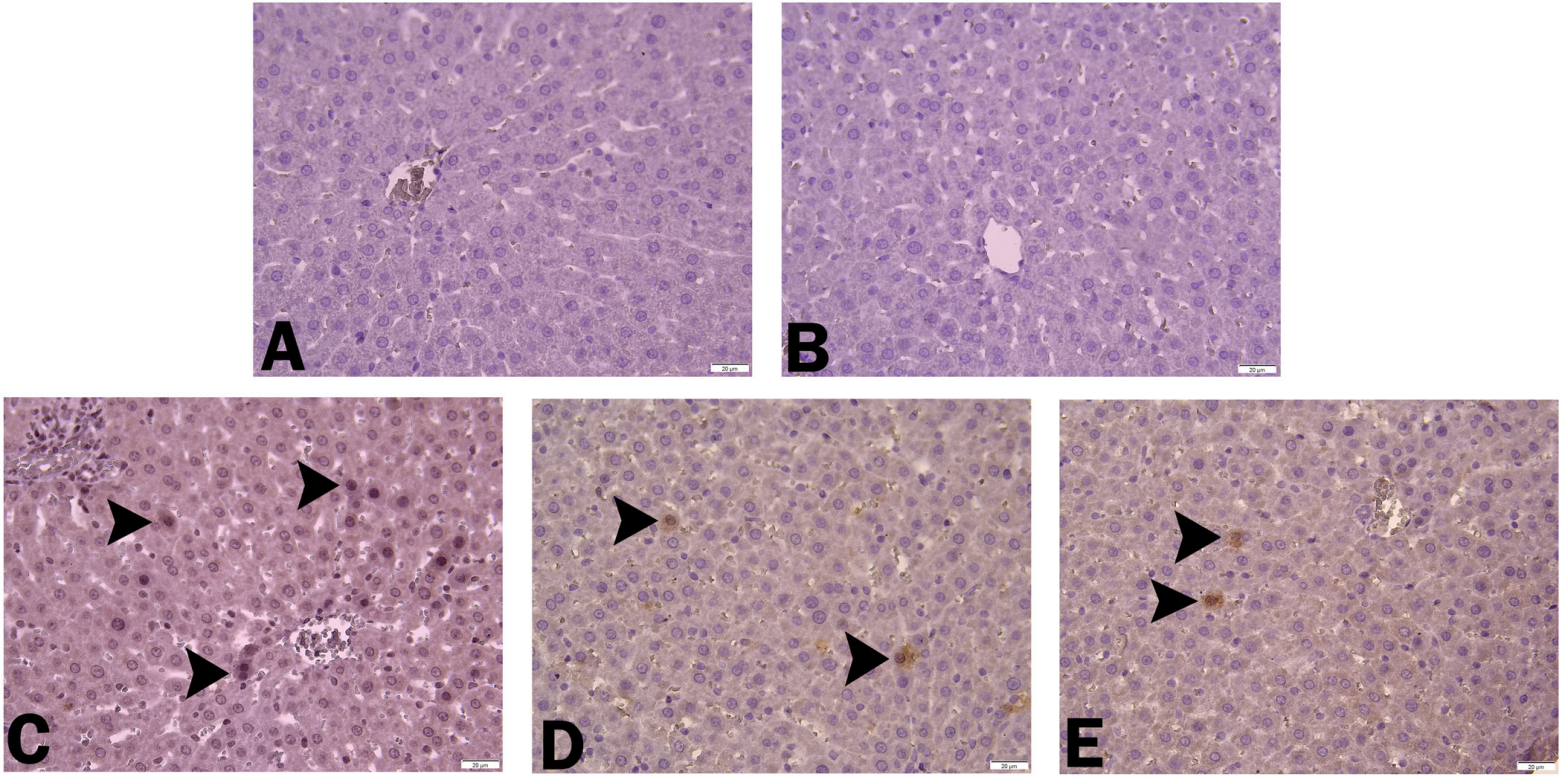
Beclin‐1 expression was negative in the liver tissue of the control (A) and CHR (B) groups. Increased beclin‐1 expression in the ACR (C) group (arrowheads). Mild beclin‐1 expression in the ACR + CHR 25 (D) and ACR + CHR 50 (E) groups (arrowheads). IHC‐P, Bar: 20 μm.

When rat liver tissues were evaluated immunohistochemically, beclin‐1 expression was seen to be negative in the livers of the control and CHR‐only given groups (Figure 5A,B). An increase in beclin‐1 expression was detected in hepatocytes in the liver tissue only in the ACR‐treated group (Figure 5C). In the ACR + CHR25 and ACR + CHR50 groups, mild beclin‐1 expression was found in hepatocytes (Figure 5D,E). Immunohistochemical findings are summarized in Table 3.

## 4 Discussion

According to the findings of the present study, ACR was found to be toxic in rat liver tissues. CHR treatment attenuated the ACR‐induced toxic effect in rat liver tissue. This effect of CHR was manifested by modulating liver function and structure, oxidative stress, inflammation, apoptosis, and parameters in significant pathways involved in cellular structure and activity.

A shift in the balance between ROS and antioxidants in tissues in favor of ROS causes oxidative stress [38]. ROS interact with lipids, proteins, and DNA in the cell, damaging their structure and ultimately causing cell damage or death [39, 40]. Oxidative stress may be the primary mechanism of tissue damage [41]. Lipid peroxidation occurs at the level of substances that react with thiobarbituric acid and hydroperoxides. On the other hand, oxidation products caused by some proteins such as carbonylated proteins are also oxidative stress damage indicators [10]. MDA, a lipid peroxidation product, is a significant indicator of oxidative stress [42, 43]. SOD, CAT, and GPx are significant antioxidant enzymes that contribute to antioxidant capacity [44]. GSH is a nonenzyme antioxidant and shows antioxidant activity by destroying ROS and detoxifying xenobiotics [45]. In the present study, in the ACR‐treated group, MDA level increased and oxidant capacity increased, GSH level and SOD, CAT, and GPx activities decreased and antioxidant capacity decreased and oxidative stress damage occurred in liver tissues. CHR tried to protect liver tissues against oxidative stress damage by reversing these effects. Similar to the present study, it has been reported that ACR significantly increased the oxidant MDA level in the liver, while decreasing the antioxidant GSH, CAT, and SOD levels in different studies [11]. Similar to the current study, Şimşek et al. [22] reported that CHR alleviated oxidant capacity by decreasing MDA level in different tissues, increased antioxidant capacity by increasing GSH level and SOD, CAT, and GPx activities and as a result, exhibited protective properties against oxidative stress damage. These findings suggest that CHR may exert its protective effects by enhancing the cellular antioxidant defense system through upregulation of SOD, CAT, GPx, and GSH, thereby neutralizing ROS and reducing oxidative damage. In addition, CHR may inhibit lipid peroxidation by reducing MDA levels, preventing membrane destabilization and cellular dysfunction. This regulatory mechanism likely contributes to restoring redox homeostasis and alleviating ACR‐induced oxidative stress injury in liver tissues.

NF‐κB is a master regulator in inflammatory diseases [46]. Increased amounts of ROS activate NF‐κB, and once activated, NF‐κB dissociates from IκB to move towards the nucleus [47]. Once transported to the nucleus, it triggers the stimulation of significant pro‐inflammatory cytokines such as NF‐κB, IL‐1β, and TNF‐α [48]. One study reported that toxicant‐induced NF‐κB dissociates from IκB and increases the release of pro‐inflammatory cytokines such as IL‐1β, TNF‐α, and IL‐6 after being transported to the nucleus [49]. TLR4 is known to play a role in pronociceptive activity together with RAGE [50]. In the present study, ACR caused inflammation damage by increasing NF‐κB, IL‐1β, TNF‐α, and TLR4 mRNA transcription levels in liver tissue. CHR protected against ACR‐induced inflammation damage by decreasing NF‐κB, IL‐1β, TNF‐α, and TLR4 mRNA transcription levels in liver tissue. Similar to the current study, Cerrah et al. [51] reported that ACR causes inflammatory damage by activating the NF‐κB pathway in liver tissues. On the other hand, studies have reported that CHR exhibits healing properties against inflammatory damage caused by different toxic agents in liver tissues by slowing down the NF‐κB pathway [22, 52, 53]. These findings suggest that CHR may exert its protective effects by inhibiting the NF‐κB signaling pathway, thereby reducing the transcription of pro‐inflammatory cytokines and suppressing TLR4‐mediated inflammatory responses. This mechanism may play an important role in alleviating ACR‐induced liver injury.

In addition, the increase in ROS may also impair posttranslational protein activity [54]. The increase in NF‐κB also affects the release of RAGE, which plays an active role in the regulation of many significant mechanisms such as cell proliferation, renewal and apoptosis, oxidative stress, and immunity [52]. NLRP3 is also activated by stimulation of exogenous factors, leading to the release of pro‐inflammatory cytokines [55]. In the present study, RAGE and NLRP3 mRNA transcription levels increased in the ACR‐treated group, increasing the interaction of different damage pathways in the tissue and causing liver tissue damage. CHR, on the other hand, provided protective properties in liver tissue by affecting this effect of ACR in the opposite direction. Consistent with the current study findings, studies have reported that CHR attenuates the inflammasome by reducing RAGE/NLRP3 activation [22, 56, 57]. These findings suggest that CHR may exert its protective effects by inhibiting the RAGE/NLRP3 signaling axis, thereby regulating inflammasome activation and reducing inflammatory damage. CHR may prevent excessive NF‐κB activation and the associated pro‐inflammatory cascade by reducing RAGE expression and ultimately alleviating oxidative stress and apoptosis. Moreover, CHR suppression of NLRP3 activation may reduce inflammation‐induced tissue damage, possibly impairing the maturation and release of pro‐inflammatory cytokines. This mechanistic interaction highlights the potential role of CHR in restoring cellular homeostasis and protecting liver tissues from ACR‐induced damage.

Matrix proteins outside the cell are rapidly degraded by metalloproteinases (MMPs). Gelatinases MMP‐2 and MMP‐9 are significant among these enzymes. Increased levels of MMP‐2 and MMP‐9 are directly related to inflammation [58]. MMPs can be directly stimulated by TNF‐α and IL‐1β. MMPs are also involved in cell proliferation, angiogenesis, and apoptosis [59]. MMP‐2, specifically secreted from hepatic stellate and Kupffer cells, provides protective properties in hepatic vascular homeostasis. MMP‐9 is an enzyme involved in the destruction of liver regeneration [60]. In the present study, MMP‐2 and MMP‐9 mRNA transcription levels increased in the ACR‐treated group. CHR attenuated this effect of ACR and decreased MMP‐2 and MMP‐9 mRNA transcription levels, thereby protecting liver tissues against damage such as inflammation and apoptosis through this pathway. Similar to this study, Yang et al. [61] reported that CHR exhibited healing properties by reducing the levels of MMP‐2 and MMP‐9. These findings suggest that CHR protects liver tissue by regulating MMP‐mediated inflammation and tissue remodeling. ACR‐induced increases in MMP‐2 and MMP‐9 likely contribute to excessive inflammation, apoptosis, and tissue damage. By reducing their expression, CHR helps maintain liver structure, prevents excessive extracellular matrix degradation, and supports tissue repair. This indicates that CHR may mitigate ACR‐induced liver injury by balancing MMP activity.

Apoptosis, which provides programmed death in cells, is an effective death pathway in the destruction of cells that need to be removed from the body. TNF‐α increase may also trigger apoptosis [62]. Apoptosis is characterized by the activation of the caspase family [63]. Apoptosis also generates cellular stress or cellular damage when it occurs excessively in healthy cells [64]. In the cell, ROS disrupts the mitochondrial membrane, increasing fluidity and permeability [65]. When the balance between Bax and Bcl‐2, two important parameters in the mitochondrial pathway, shifts in favor of Bax, cytochrome C is released from the mitochondria into the stasis and caspase activation begins [66]. Mainly the increase in ROS increases Casp‐3 activation [67]. Casp‐3 is the most significant caspase and is known as the executioner caspase. Casp‐3 is the irreversible key point in cell death [68]. Casp‐9 is the caspase family member that activates Casp‐3 [69]. In a study conducted with different toxic agents, it was reported that the amount of cytochrome C increased in liver tissue as a result of Bax/Bcl‐2 balance which was disrupted with increasing oxidative stress and apoptosis occurred as a result of Casp‐3 activation [70]. In the present study, ACR administration disrupted the Bax/Bcl‐2 balance in favor of Bax, leading to Casp‐6, Casp‐9, and Casp‐3 activation and consequently apoptotic damage. CHR reversed this situation and protected liver tissue against ACR‐induced apoptotic damage. These findings suggest that CHR may exert its protective effects by regulating the intrinsic (mitochondrial) apoptotic pathway. By restoring the Bax/Bcl‐2 balance in favor of Bcl‐2, CHR may inhibit caspase cascade activation, possibly preventing mitochondrial membrane instability and cytochrome c release. Suppression of Casp‐9 and Casp‐3 activation by CHR suggests its role in preventing excessive apoptotic signaling and preserving cellular integrity. In addition, the antioxidant properties of CHR may contribute to reducing ROS‐mediated mitochondrial dysfunction and further attenuating apoptosis.

ACR‐induced tissue apoptosis also involves cellular autophagy [71]. Beclin‐1 is one of the critical indicators of autophagy. Beclin‐1 is active in tumor suppression and in the development of the immune system [72]. In the present study, beclin‐1 activity was examined immunohistochemically, and ACR‐induced increase in liver tissue was observed. CHR protected liver tissue against ACR‐induced autophagic damage by decreasing beclin‐1 activity. Triningsih et al. [71] reported that ACR increased cellular beclin‐1 levels. On the other hand, Tuncer et al. [73] reported that CHR exhibited ameliorative properties against autophagic damage by reducing Beclin‐1 expression induced by different agents. These findings suggest that CHR may exert its protective effects by modulating the autophagic pathway through the downregulation of Beclin‐1 expression. The increase in beclin‐1 levels induced by ACR suggests an excessive or dysregulated autophagic response, which may contribute to cellular stress and liver tissue damage. CHR's ability to reduce beclin‐1 activity indicates a potential role in restoring autophagic balance, preventing excessive autophagy‐related apoptosis, and maintaining cellular homeostasis. This mechanism highlights CHR's regulatory influence on the interplay between autophagy and apoptosis in ACR‐induced liver injury.

ALT, AST, and ALP activities are markers of liver function [74]. As liver toxicity worsens, liver failure may occur and consequently, liver transplantation may be required [75]. Toxic injury results in damage to hepatocytes and activation of Kupffer cells and natural killer cells, resulting in an inflammatory reaction [76]. In the present study, ACR impaired liver function and caused an increase in ALT, AST, and ALP activities. When CHR was administered together with ACR, ALT, AST, and ALP activities were reversed and CHR provided protective properties. Consistent with the current study, studies [12, 51] reported that ACR increased ALT and AST activities in liver tissues. This was demonstrated in H&E imaging of liver tissue. In the liver tissue images of the ACR‐treated group, severe necrosis, hydropic degeneration, mononuclear cell infiltration, sinusoidal dilatation, severe hyperemia, and hemorrhage in vessels and sinusoids were observed in hepatocytes. In the experimental groups in which CHR was administered together with ACR, it was determined that CHR exhibited significant protective properties against ACR‐induced damage in liver tissue. Kandemir et al. [53] reported that CHR reduced the increased ALT, AST, and ALP activities resulting from liver toxicity caused by different toxic agents and alleviated tissue damage.

## 5 Conclusion

As a conclusion, the toxic effect of ACR, which we are commonly exposed to from many food sources in our daily lives, on rat liver tissues and the protective effect of CHR were determined by different methods and different damage pathways. CHR exhibited protective properties by alleviating ACR‐induced oxidative stress, DNA damage, inflammation, apoptosis, autophagy, and disorders in tissue function and structure in rat liver tissues.

## 6 Limitations of the Study

This study has some limitations. Since a rat model was used, the findings are limited to direct generalization to human physiology. A specific dose and duration were used, and different doses and long‐term effects were not evaluated. Given these limitations, alternative studies are also needed.

## Author Contributions

**Selman Gencer:** research, methodology, investigation, wrote the first draft of the article. **Hasan Şimşek:** methodology, investigation, data curation, formal analysis, wrote the first draft of the article. **Cihan Gür:** methodology, investigation, data curation, formal analysis. **Mustafa İleritürk:** methodology, investigation, data curation, formal analysis. **Sefa Küçükler:** methodology, investigation, data curation, formal analysis. **Nurhan Akaras:** histopathological examination. **Fatih Mehmet Kandemir:** investigation, formal analysis, supervision. All authors read and approved the final manuscript.

## Ethics Statement

Ethical approval was obtained from Necmettin Erbakan University Local Animal Experiments Ethics Committee (22.12.2023, 2023–54).

## Consent

The authors gave their explicit consent to publish the manuscript. Selman Gencer is free to contact any of the people involved in the research to seek further clarification and information.

## Conflicts of Interest

The authors declare no conflicts of interest.

## Data Availability

Data will be made available on request.
